# Experimental data on activity catalyst TiO_2_/Fe_3_O_4_ under natural solar radiation conditions

**DOI:** 10.1016/j.dib.2020.105490

**Published:** 2020-04-19

**Authors:** A. Castro-Sánchez, R. Camargo-Amado, F. Machuca-Martínez

**Affiliations:** Escuela de Ingeniería Química, Universidad del Valle, Calle 13 No. 100-00, Cali, Colombia

**Keywords:** Dye, Catalyst support, Compound parabolic collector, Pilot plant, Magnetite, TiO_2_

## Abstract

In this document, the photocatalytic activity of TiO_2_/Fe_3_O_4_, prepared by the mixing of the pure oxides, was studied. The photocatalytic degradation of aqueous Methylene Blue (MB) solutions (10 and 30 ppm) was performed, the TiO_2_/Fe_3_O_4_ catalysts in 80/20, 50/50 and 20/80 mass ratios were used during the test, artificial sunlight and natural solar radiation were tested at laboratory and pilot plant scale respectively. Besides, the kinetic reactions were evaluated according to the Langmuir-Hinshelwood model, the apparent velocity constants (k_app_) were obtained for the TiO_2_/Fe_3_O_4_ catalysts.

In the laboratory test, the TiO_2_/Fe_3_O_4_ catalyst (80/20) had a performance for 93.04% of discoloration, k_app_ = 0.0238 min^−1^, while for TiO_2_/Fe_3_O_4_ (50/50, 20/80) had an 83.46%, 65.00% for discoloration of MB and the k_app_ values were 0.0154 min^−1^ and 0.0098 min^−1^, respectively.

In the solar test at pilot scale, the percentages of discoloration of 24.32%, and 57.78%, with k_app_ values of 0.00037 min^−1^, 0.00121 min^−1^ respectively were obtained for TiO_2_/Fe_3_O_4_ (80/20), a MB solution of 30 ppm, a load of 0.1 g/L and 0.3 g/L of the catalyst respectively.

Specifications table

**Table:** 

Subject	Chemical Engineering
Specific subject area	Advanced Oxidation Processes
Type of data	Tables and Figures
How data were acquired	The TiO_2_/Fe_3_O_4_ materials were analysed by FT-IR, the raw data of degradation and accumulated energy was obtained by UV–vis spectrophotometry, and radiometry UV respectively.
Data format	Raw and analysed
Parameters for data collection	All experimental tests were carried out to laboratory and pilot scale. A batch type photoreactor was used under the irradiation provided by a xenon lamp in a solar simulator, with a light intensity of 150 klx (kilolux), radiation intensity of 500 W/m^2^ and with a cooling system. Pilot-scale, CPC reactor was used exposed under sunlight, following the reaction up to 100 kJ/m^2^
Description of data collection	TiO_2_/Fe_3_O_4_ was prepared by a process of mixing the pure oxides in mass ratios of 80%, 50% and 20% of TiO_2_, the photocatalytic activity was evaluated by the discoloration of MB. Laboratory scale teste were made with catalysts at 10 and 30 ppm MB in batch reactors under a SUNTEST system for 2 h. After 30 min of adsorption-desorption equilibrium, the first sample was taken, then the lamp was turned on and a sample of the first half hour was taken every 10 min; then at 60 min and finally at 120 min. The absorbances of the samples were taken and the MB color concentration was known. The best performing catalyst in the first stage were selected for sunlight testing. These tests were carried out on a pilot under natural solar radiation with 10 and 30 ppm MB solutions in a compound parabolic collector (CPC) reactor, and showing every 10 kJ/m^2^ up to 100 kJ/m^2^ of accumulated energy.
Data source location	Universidad del Valle, A.A. 25,360, Calle 13 No. 100–00 Cali, Colombia.
Data accessibility	The data is found only in this article.

## Value of the data

• The TiO_2_/Fe_3_O_4_ is a useful catalyst because its photocatalytic and magnetic properties allow the degradation of dye and then it can later be removed easily
• The data can be used to model reactors under real conditions.
• The information recorded here is important in the work of innovating in profitable and sustainable catalysts in its industrial application for wastewater treatment.

## 1 Data

The data describes the obtaining of the TiO_2_/Fe_3_O_4_ catalyst and its use in the degradation of Methylene Blue (MB) solutions by photocatalysis. The mixing catalyst was prepared easily with promising functionality due to the excellent data obtained from the degradation of the model pollutant and its ease to remove it from the remaining medium [1], [2], [3], [4].

**Fig. 1:**
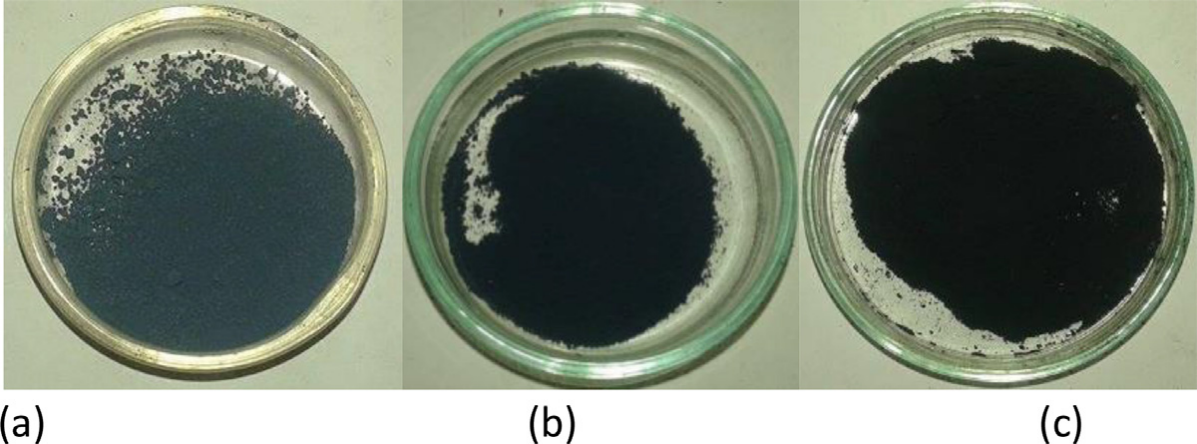
Synthesized TiO_2_/Fe_3_O_4_ catalysts (a) 80/20; (b) 50/50; (c) 20/80.

**Fig. 2:**
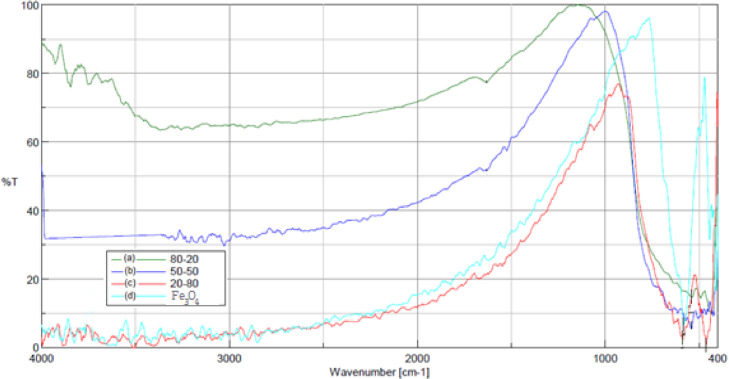
FT-IR spectrum (a) TiO_2_/Fe_3_O_4_ (80/20) (b) TiO_2_/Fe_3_O_4_ (50/50), (c) TiO_2_/Fe_3_O_4_ (20/80), (d) Fe_3_O_4_.

**Fig. 3:**
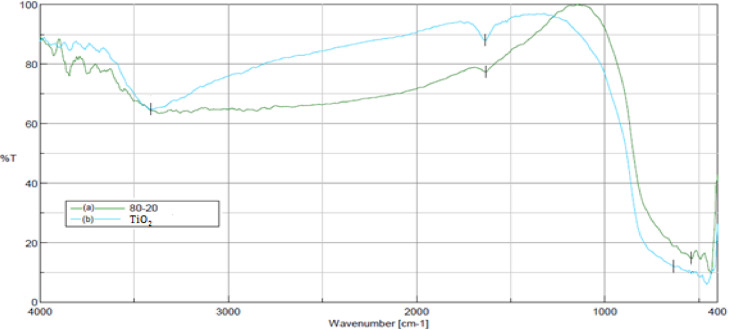
FT-IR spectrum (a) TiO_2_/Fe_3_ (80/20), (b) pure TiO_2_.

Fig. 1 shows the photocatalysts of TiO_2_/Fe_3_O_4_ with different proportions of the oxides. Fig. 2 illustrates the infrared spectra of the synthesized catalysts and Fe_3_O_4_, as well as in Fig. 3 with the FT-IR spectra of TiO_2_/Fe_3_O_4_ (80/20) and pure TiO_2_.

**Fig. 4:**
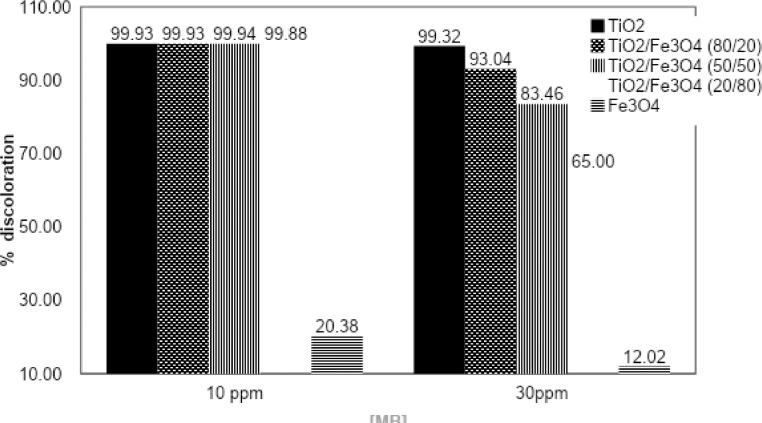
MB discoloration under simulated solar reactor by catalysts of TiO_2_, TiO_2_/Fe_3_O_4_ and Fe_3_O_4_. [catalyst] = 0.3 g/L.

**Fig. 5:**
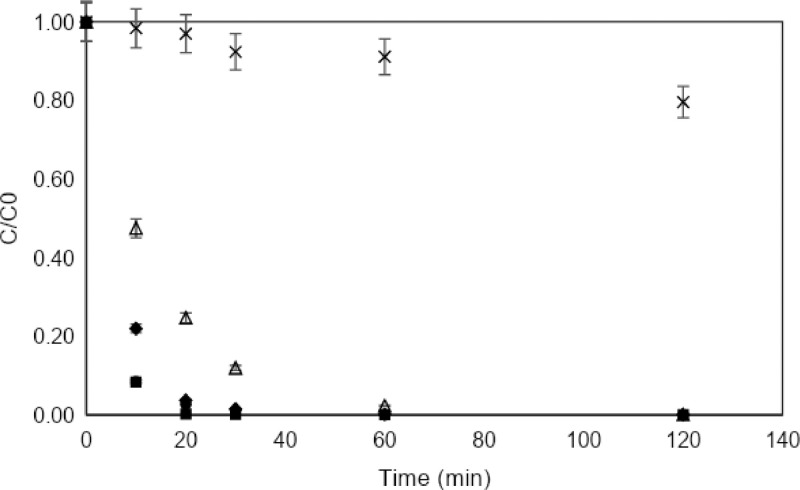
Discoloration of MB under solar simulator at lab test [MB] = 10 ppm, [catalyst]=0.3 g/L. (■) TiO_2_, (●) TiO_2_/Fe_3_O_4_ (80/20), (♦) TiO_2_/Fe_3_O_4_ (50/50), (∆) TiO_2_/Fe_3_O_4_ (20/80), (×) Fe_3_O_4_.

**Fig. 6:**
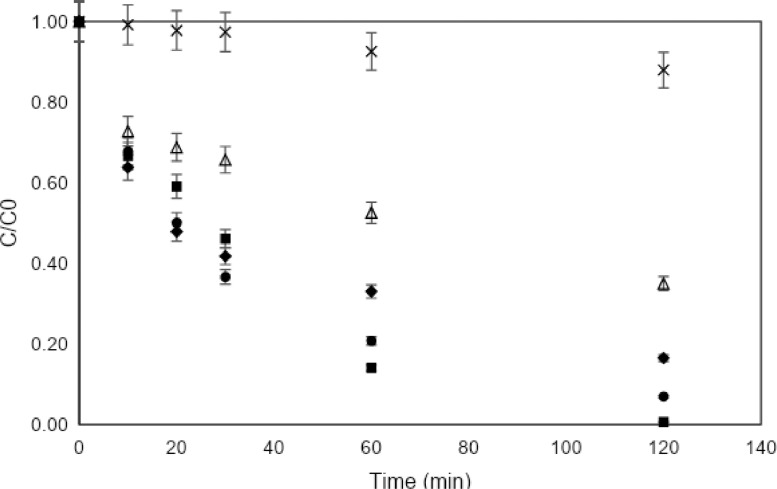
Discoloration of MB under solar simulator at lab test [MB] = 30 ppm, [catalyst]=0.3 g/L. (●) TiO_2_/Fe_3_O_4_ (80/20), (♦) TiO_2_/Fe_3_O_4_ (50/50), (∆) TiO_2_/Fe_3_O_4_ (20/80), (×) Fe_3_O_4_.

Fig. 4 compares the discoloration achieved in laboratory tests with the photocatalysts that were prepared. Fig. 5, Fig. 6 illustrate the variation in the degradation of MB according to the initial dye concentration 10 ppm and 30 ppm, respectively.

**Fig. 7:**
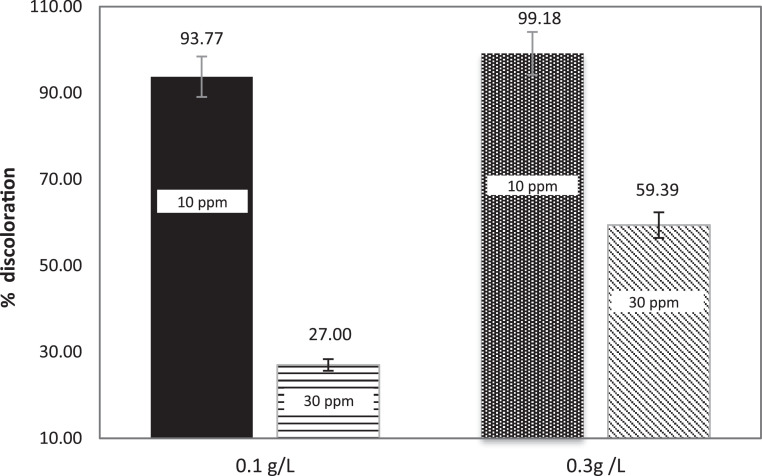
Comparison of MB discoloration by of TiO_2_/Fe_3_O_4_ (80/20) under natural solar test at pilot scale. [TiO_2_/Fe_3_O_4_] = 0.1 g/L and 0.3 g/L.

**Fig. 8:**
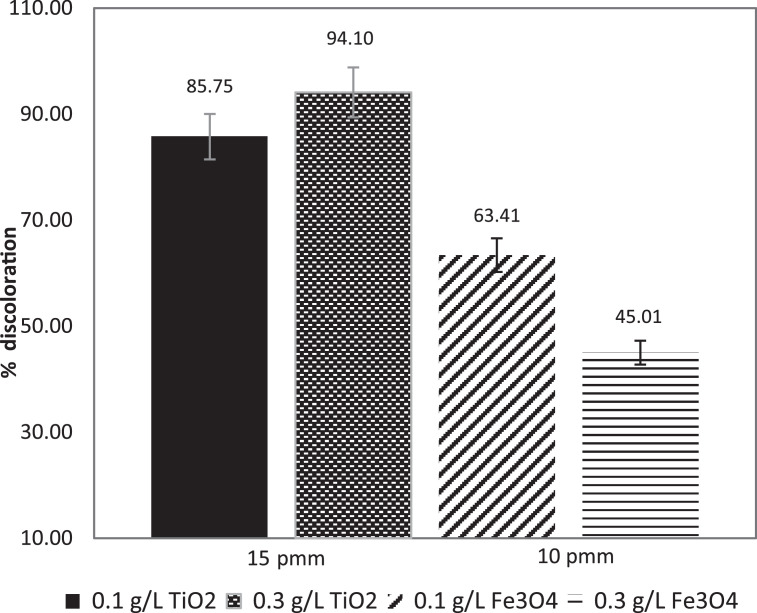
Comparison of MB discoloration under natural solar test at pilot scale, by of TiO_2_ and Fe_3_O_4_ [catalysts] = 0.1 g/L and 0.3 g/L.

The discoloration levels of MB solutions in a CPC solar reactor with accumulated energy of 100 kJ/m^2^ for different concentrations of TiO_2_/Fe_3_O_4_ (80/20), TiO_2_ and Fe_3_O_4_ respectively, are compared in Fig. 7, Fig. 8.

**Fig. 9:**
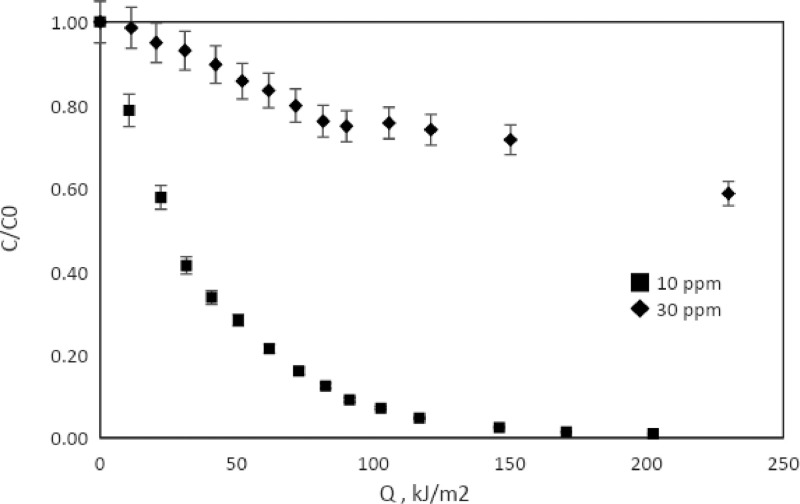
Degradation of AM vs UV energy accumulated. 0.1 g/L of TiO_2_/Fe_3_O_4_ (80/20).

**Fig. 10:**
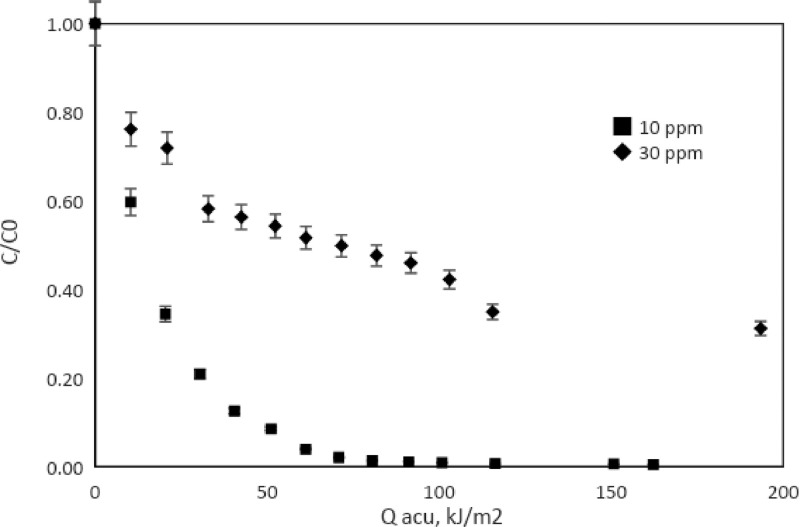
Degradation of AM vs UV energy accumulated. 0.3 g/L of TiO_2_/Fe_3_O_4_(80/20).

Table 1 shows the adsorption values of the catalysts for a 30 ppm methylene blue solutions. Table 2 and Table 3 shows the percentages of discoloration and the rate constant for reactions at laboratory scale and pilot scale. Finally, Fig. 9, Fig. 10 present the monitoring of the reaction (C/Co) as a function of the accumulated solar energy for two concentrations of MB with the TiO_2_/Fe_3_O_4_ (80/20), 0.1 g/L and 0.3 g/L loads, in your order.

**Table 1:** Adsorption percentage of catalysts on dark stage, [MB]= 30 ppm, [catalyst]= 0.3 g/L.

Catalyst	% Adsorption
TiO_2_	10.19
TiO_2_/Fe_3_O_4_ (80/20)	17.15
TiO_2_/Fe_3_O_4_ (50/50)	16.07
TiO_2_/Fe_3_O_4_ (20/80)	10.97
Fe_3_O_4_	0.70

**Table 2:** Percent discoloration of MB under simulated solar radiation (120 min), and the apparent constant of the first order (k_app_), [catalysts] = 0.3 g/L.

	10 ppm		30 ppm	
Catalysts	% discoloration	k_app_ (min^−1^)	% discoloration	k_app_ (min^−1^)
TiO_2_	99.93	0.0828	99.32	0.0389
TiO_2_/Fe_3_O_4_ (80/20)	99.93	0.0768	93.04	0.0238
TiO_2_/Fe_3_O_4_ (50/50)	99.94	0.0767	83.46	0.0154
TiO_2_/Fe_3_O_4_ (20/80)	99.88	0.0585	65.00	0.0098
Fe_3_O_4_	20.38	0.0019	12.02	0.0011

**Table 3:** Percent discoloration of MB under natural solar radiation and the average apparent first order rate constant (k_app_) with 100 kJ/m^2^ accumulated energy.

	[catalysts] = 0.1 g/L			
	10 ppm		30 ppm	
	% discoloration	k_app_ (m^3^/kJ)	% discoloration	k_app_ (m^3^/kJ)
TiO_2_	99.05	0.00854	71.57	0.00132
TiO_2_/Fe_3_O_4_ (80/20)	92.86	0.00318	24.32	0.00037

	[catalysts] = 0.3 g/L			
TiO_2_	99.02	0.00693	89.93	0.00261
TiO_2_/Fe_3_O_4_ (80/20)	99.11	0.00626	57.78	0.00121

## 2 Experimental design, materials, and methods

### 2.1 Material

Percentages of 20, 50 and 80% by weight of TiO_2_ (AEROXIDE® TiO_2_ P-25, Evonik) and Fe_3_O_4_ (iron oxide NP (II, III), Sigma-Aldrich) were used for the synthesis of TiO_2_/Fe_3_O_4_ catalysts; solutions of NaOOCCH_3_•3H_2_O (sodium acetate trihydrate, ACS Fisher Scientific) and CH_3_COOH (commercial acetic acid) were also used to adjust the pH; Methylene Blue solutions, analytical grade (Mol Labs), were prepared as a model contaminant.

### 2.2 Photocatalytic reactors

For the development of the present investigation, a batch type photoreactor was used, this is a Pyrex type beaker, covered with a Petri dish to avoid evaporation of the MB solutions due to the radiation of the xenon lamp in a solar simulator, ORIGINAL HANAU SUNTEST. In this system, the lamp is located approximately 10 cm from the reactor, the light intensity is 150 klx (kilolux), radiation intensity of 500 W/m^2^. Additionally, the system is provided with recirculation of water around and ventilation (60m^3^/h), to avoid heating the solutions above 45 °C.

The Compound Parabolic Collector (CPC) type solar reactor was used for pilot and solar scale tests. This reactor consists of 6 borosilicate glass tubes, connected in series mounted on the reflective aluminum collector surface that distributes direct and diffuse solar radiation; each tube has a length of 1.5 m, a diameter of 0.025 m and 1.4 mm thick, which comprises a total irradiated area of 0.225m^2^. The hydraulic system of this reactor operates with a centrifugal pump that recirculates the fluid through the irradiated section of the tubes to the recirculation and mixing tank. The flow rate provided was approximately 24 Lmin-1, which ensures a turbulent flow (*Re* > 10000), with a volume of 28 L.

### 2.3 Experimental

3.5 g of the TiO_2_/Fe_3_O_4_ catalysts were prepared in percentages of 20, 50 and 80% by weight of TiO_2_. Thus, for the TiO_2_/Fe_3_O_4_ catalyst 20% by weight of TiO_2_, 0.7 g of TiO_2_ and 2.8 g of Fe_3_O_4_ were taken for the 20% by weight catalyst of TiO_2_ and dispersed separately in 200 ml of filtered water. Each suspension was adjusted to pH 6 with NaOOCCH_3_•3H_2_O and CH_3_COOH solutions, taken to an ultrasound bath (Elma-Transsonic TS 540) for 2 min to achieve a good dispersion of each oxide and mixed in equal volumetric proportions. This mixture was also taken to the ultrasound bath for 6 min, then filtered with a Millipore microfiltration membrane (0.45 μm) in a polysulfone vacuum filtration funnel. The solid obtained was removed and allowed to dry in the convection drying oven at 60 °C, to finally macerate to obtain a powder of very fine particles for testing [5]. This procedure was followed to obtain TiO_2_ catalysts at 50% and 80% by weight.

For laboratory experiments with artificial sunlight [6], solutions of 10 and 30 ppm of analytical grade MB (Mol Labs) in filtered water were prepared to perform 300 mL discoloration tests of these MB solutions in the photoreactor. The experiments were performed for each concentration of MB with 0.3 g of each of the three catalysts prepared (TiO_2_/Fe_3_O_4_ 20/80, 50/50 and 80/20) and with 0.3 g for the targets, corresponding to TiO_2_ and Fe_3_O_4_; they were suspended by stirring (mechanical) at natural pH of the solutions (7.3 - 7.5). Stirring was done in the dark for 30 min before irradiation to reach the adsorption-desorption equilibrium of the dye on the catalyst [7]. After 30 min of equilibrium, the first 3 mL sample was taken with a syringe, then the lamp was turned on and the first half hour was sampled every 10 min; then at 60 min and finally at 120 min of having started lighting. At all times 3 mL of sample was taken, and at the end of the process the volume in the reactor was 285 mL.

The experiments on a solar scale began with the preparation of the 10 ppm and 30 ppm MB solutions in the mixing tank, according to the volume of work and then the pump was put into operation to facilitate mixing and achieve a flow with an initial concentration in steady state; After 3 min a sample was taken, the catalyst was added and the tank was closed. During this preparation and for a time of 30 min, from the addition of the catalyst (in concentrations of 0.1 g/L and 0.3 g/L), the photo-reactor is protected with a black cover to prevent the reaction from starting earlier that the substrate adsorption equilibrium is reached on the catalyst surface [8]. After 30 min in which the adsorption-desorption equilibrium is guaranteed, a sample of the circulating solution was taken and the reactor was discovered, and from that moment the process of solar discoloration was followed by measuring the accumulated energy until reaching a value of 100 kJ/m^2^ as the standard solar irradiance, by means of the radiometer and the UV-A probe located near and in the same position of the reactor [9], every 10 kJ/m^2^ up to 100 kJ/m^2^ samples were taken of the fluid that exits the tubes from the reactor and returned to the mixing tank.

### 2.4 Analytical techniques

Infrared Spectroscopy (Jasco/4100–Jasco Corporation FT-IR spectrometer, transmittance with KBr tablet) was used to observe some of the structural characteristics of the synthesized catalysts. To obtain the spectrum of the MB dye, a UV–VIS spectrophotometer (SpectroQuant Merck Pharo 300) was used and through the same technique the absorbency was measured in relation to the color of the samples taken in the experiments. The energy accumulated in the solar-scale experiments was monitored using a photo-radiometer (Delta Ohm-HD2102.2, with LP 471 UVA probe) to track the system in the time of solar exposure that affects the surface of the system. In addition, a portable DO dissolved oxygen meter (5-Star Plus Thermo Scientific Orion, with DO probe) was used.

## Appendix Supplementary materials

Image, application 1

Image, application 2
